# Association of BRM promoter polymorphisms and esophageal adenocarcinoma outcome

**DOI:** 10.18632/oncotarget.15890

**Published:** 2017-03-03

**Authors:** Grzegorz J. Korpanty, Lawson Eng, Xin Qiu, Olusola Olusesan Faluyi, Daniel J. Renouf, Dangxiao Cheng, Devalben Patel, Zhuo Chen, Brandon C. Tse, Jennifer J. Knox, Lorin Dodbiba, Jennifer Teichman, Abul Kalam Azad, Rebecca Wong, Gail Darling, David Reisman, Sinead Cuffe, Geoffrey Liu, Wei Xu

**Affiliations:** ^1^ Princess Margaret Cancer Centre, Department of Medicine, University Health Network, Toronto, ON, Canada; ^2^ Canadian Cancer Trials Group, Department of Medicine, Queens University, Kingston, ON, Canada; ^3^ Princess Margaret Cancer Centre, Department of Biostatistics, University Health Network, Toronto, ON, Canada; ^4^ Department of Medical Oncology, University of British Columbia and British Columbia Cancer Agency, Vancouver, BC, Canada; ^5^ Princess Margaret Cancer Centre, Department of Medical Biophysics, University Health Network, Toronto, ON, Canada; ^6^ Princess Margaret Cancer Centre, Radiation Medicine Program, University Health Network, Toronto, ON, Canada; ^7^ Department of Surgery, Division of Thoracic Surgery, Toronto General Hospital, University Health Network, Toronto, ON, Canada; ^8^ Department of Medicine in the College of Medicine, Division of Hematology and Oncology, University of Florida, Gainesville, FL, USA; ^9^ Department of Epidemiology, Dalla Lana School of Pubic Health, Toronto, ON, Canada

**Keywords:** chromatin remodeling, polymorphism, cancer prognosis, esophageal cancer, brahma

## Abstract

**Purpose:**

Brahma (BRM) is a critical catalytic subunit of the SWI/SNF chromatin remodeling complex; expression of BRM is commonly lost in various cancer types. BRM promoter polymorphisms (BRM-741; BRM-1321) are associated with loss of BRM expression, and with cancer risk/survival. We evaluated these two polymorphisms in the overall survival (OS) of esophageal adenocarcinoma (EAC) patients.

**Results:**

Of 270 patients, 37% were stage IV. Minor allele frequencies were 47−49%; 15% were double-homozygotes. When compared to the wild-type genotype, the homozygous variant of BRM-741 carried an adjusted OS hazard ratio (aHR) of 1.64 (95% CI:1.1−2.4); for BRM-1321, the aHR was 2.09 (95% CI:1.4−3.0). Compared to the double wild-type, carrying homozygous variants of both promoter polymorphisms (double-homozygote) yielded an aHR of 2.21 (95% CI:1.4−3.6). Directions/magnitudes of associations were similar in subsets by age, gender, smoking status, use of platinum agents, and disease stage, and for progression-free survival.

**Materials and Methods:**

In a cohort of EAC patients of all stages (84% male; median age of 64 years), two BRM polymorphisms were genotyped. Cox proportional hazards models, adjusted for known prognostic variables, estimated the association of polymorphisms with OS.

**Conclusions:**

BRM polymorphisms were associated with OS in EAC in this study. Validation studies are warranted.

## INTRODUCTION

Esophageal adenocarcinoma (EAC) incidence has risen dramatically in recent years in North America and other Western countries, yet the reason for this remains largely unknown. [1–4] However, chronic, symptomatic gastroesophageal reflux, which is associated with Barrett's metaplasia and dysplasia, is now a recognized risk factor for EAC. [5] Esophageal cancer continues to have poor prognosis, with metastatic disease having a 5-year overall survival of under one percent, while the 5-year rate of survival in patients who have undergone definitive treatment ranges from 5%-30% (~15%) [6].

Exome and whole-genome sequencing studies have identified several significantly mutated genes in both squamous cell esophageal carcinoma and EAC. [7–9] In addition to genes that were previously implicated in EAC such as *TP53*, *SMAD4*, *CDKN2A PIK3CA*, and *ARID1A*, significantly mutated genes in 24% of tumours have included genes that code for chromatin-modifying proteins such as *SMARCA4*, a key member of the SWI/SNF family. [7] SWI/SNFs (SWItch/sucrose non-fermentable), a highly related family of multi-subunit complexes, are chromatin remodeling enzymes that are both ATP-dependent and involved intimately in the regulation of multiple functions, including differentiation, development, gene expression, cell adhesion, DNA repair and cell cycle control. [10] SWI/SNFs mediate gene expression through the process of shifting histone positions, thus having parts of the DNA made more or less accessible to important cellular regulatory proteins such as transcription factors. BRCA1, p53 and retinoblastoma (Rb) proteins are examples of signal transduction pathways and anticancer proteins that are dependent functionally on this complex. [11, 12] The mammalian SWI/SNF complexes are composed of: (1) either brahma homologue (SMARCA2 or BRM) or BRG1/SMARCA4 (BRM/SWI2-related gene 1), which are mutually exclusive catalytic ATPase subunits; (2) a set of core, highly conserved subunits including SNF5 (also known as SMARCB1, INI1 and BAF47), BAF155 and BAF170); (3) and variant subunits involved in the assembly, regulation and targeting of complex-related lineage-specific functions. [10, 13] in 15–20% of many different types of solid tumors, *BRM* is silenced, and after exposure to carcinogens, there is a ten-fold increase in tumor development in *BRM*-deficient mice. A conclusion of these data is that there is a high likelihood that *BRM* is a cancer susceptibility gene, [14] where in the presence of other environmental factors, *BRM* loss may promote carcinogenesis. Further, loss of heterozygosity (LOH) at the *BRM* locus at 9p23–24 is observed commonly in many different tumor types. [15–18] Two novel *BRM* insertion promoter polymorphisms, *BRM −1321* and *BRM −741*) are correlated with the epigenetic silencing of *BRM* (through recruitment of histone deacetylases) and increased risk of lung, head and neck, and upper digestive tract cancers. [19–21] It also has been reported recently that *BRM −1321* and *BRM −741* homozygous variants are strongly associated with adverse survival in pancreatic cancers. [22] Reversing the pharmacological effects can lead to epigenetic silencing; these are changes to BRM that form a potentially viable treatment strategy, which makes BRM an interesting subject of translational research [23].

Here we report association of *BRM* promoter polymorphisms (*BRM −1321* and *BRM −741*) with the survival of esophageal adenocarcinoma patients.

## RESULTS

Of 653 esophageal cancers identified during this period retrospectively by the cancer registry, only 374 were deemed eligible. Main reasons for ineligibility included reclassification into gastroesophageal adenocarcinoma Siewert III or gastric adenocarcinoma, squamous cell histology, second opinions, anticipated survival less than 3 months or deemed ineligible for any treatment after diagnosis for any reason (e.g. kidney failure, etc.). Of these, 323/374 (86%) patients had successful contact with the study coordinator, 282/323 (87% of those approached) provided consent, and 270/282 (96% of those consented) completed the epidemiological data, were actually treated at PM, and provided blood samples for analysis. 268/270 (99%) samples had passed quality control for *BRM-741* genotyping, while 265/270 (98%) passed control for *BRM-1321* genotyping. There was linkage disequilibrium between the two polymorphisms in this dataset (D’= 0.83).

Most patients were Caucasians (87%), males (84%), ex- or current smokers (74%) and presented with non-metastatic (resectable) disease (65%). Almost all patients had Eastern Cooperative Oncology Group (ECOG) performance status of 0–1 (94%). Patient characteristics are presented in Table 1. After a median follow up of 58.5 months, median OS time was 20.8 (95% CI: 17.4–26.2) months and median PFS time of 12.9 months (95% CI: 10.7–15.9) for the entire cohort. In the univariable analysis, advanced TNM classification, lack of presence of Barrett's esophagus, lack of surgery and radiotherapy as first line of treatment were associated with worse OS (Table 2), and the resultant clinical base model incorporated the following variables: age, ECOG, weight loss (%), TNM Classification and surgical resection.

**Table 1:** Patient demographics and clinical characteristics (*N* = 270)

Variable	Categories	N (%)
Ethnicity	Caucasian	235 (87)
	Other	35 (13)
Sex	Male	228 (84)
	Female	42 (16)
Age	Median, range (years)	64.1 (29–88)
Smoking status > 100 cigarettes in one's lifetime	Never-smokers	71 (26)
	Former smokers	138 (51)
	Current smokers	61 (23)
Alcohol consumption > 1 drink/month in any one year	Yes	220 (82)
	No	50 (18)
Performance status (ECOG)	0	58 (21)
	1	196 (73)
	2–3	16 (6)
Presence of weight loss at diagnosis	< 10%	147 (54)
	≥ 10%	122 (45)
	Not reported	1 (0)
TNM stage	1	29 (11)
	2	73 (27)
	3	73 (27)
	4	95 (35)
Tumour location	Proximal/Middle esophagus	15 (6)
	Distal esophagus	124 (46)
	Gastro-esophageal junction*	131 (48)
Presence of Barrett's esophagus	Yes	45 (17)
	No	225 (83)
Surgical Resection Performed as part of first line therapy	Yes	172 (64)
	No	98 (36)
Chemotherapy given as part of first line therapy	Cisplatin-based chemotherapy	167 (62)
	Other chemotherapy	11 (4)
	No	92 (34)
Primary radiotherapy given as part of first line therapy	Yes	152 (56)
	No	118 (44)

*Siewert classification of gastroesophageal cancers: I and II only.

**Table 2:** Univariate analysis of demographic and clinical factor with overall survival

Variables	Comparisons	Overall Survival		
		HR	95% CI	*p*-value
Ethnicity	Non-Caucasian vs Caucasian	1.01	0.59–1.72	0.96
Sex	Female vs Male	0.69	0.46–1.03	0.072
Age	Per increase in one year	1.0	0.99–1.02	0.46
Alcohol intake	Yes vs No	1.29	0.89–1.29	0.18
Smoking	Ever vs Never	1.04	0.76–1.04	0.80
Weight loss	< 10% vs ≥ 10%	0.61	0.47–0.81	0.0004
	per one percent increase in weight loss	0.99	0.99–0.99	< 0.0001
Barrett's esophagus	Yes vs No	0.67	0.45–0.98	0.037
Performance Status*	ECOG 1 vs 0	1.05	0.75–1.47	0.77
	ECOG 2 or 3 vs 0	2.19	1.12–4.01	0.011
Disease stage at diagnosis**	Stage 2 vs 1	1.94	1.09–3.46	0.024
	Stage 3 vs 1	3.51	1.98–6.23	0.0003
	Stage 4 vs 1	5.31	3.02–9.35	< 0.0001
Surgical resection	Yes vs No	0.26	0.19–0.34	< 0.0001
Chemotherapy as first line of treatment	Yes vs No	1.20	0.89–1.61	0.22
Treatment with cisplatin chemotherapy as first line	Yes vs No	1.15	0.87–1.53	0.32
Radiotherapy as first line of treatment	Yes vs No	1.42	1.07–1.87	0.014

*Global *p*-value = 0.007; **Global *p*-value < 0.0001.

**Figure 1:**
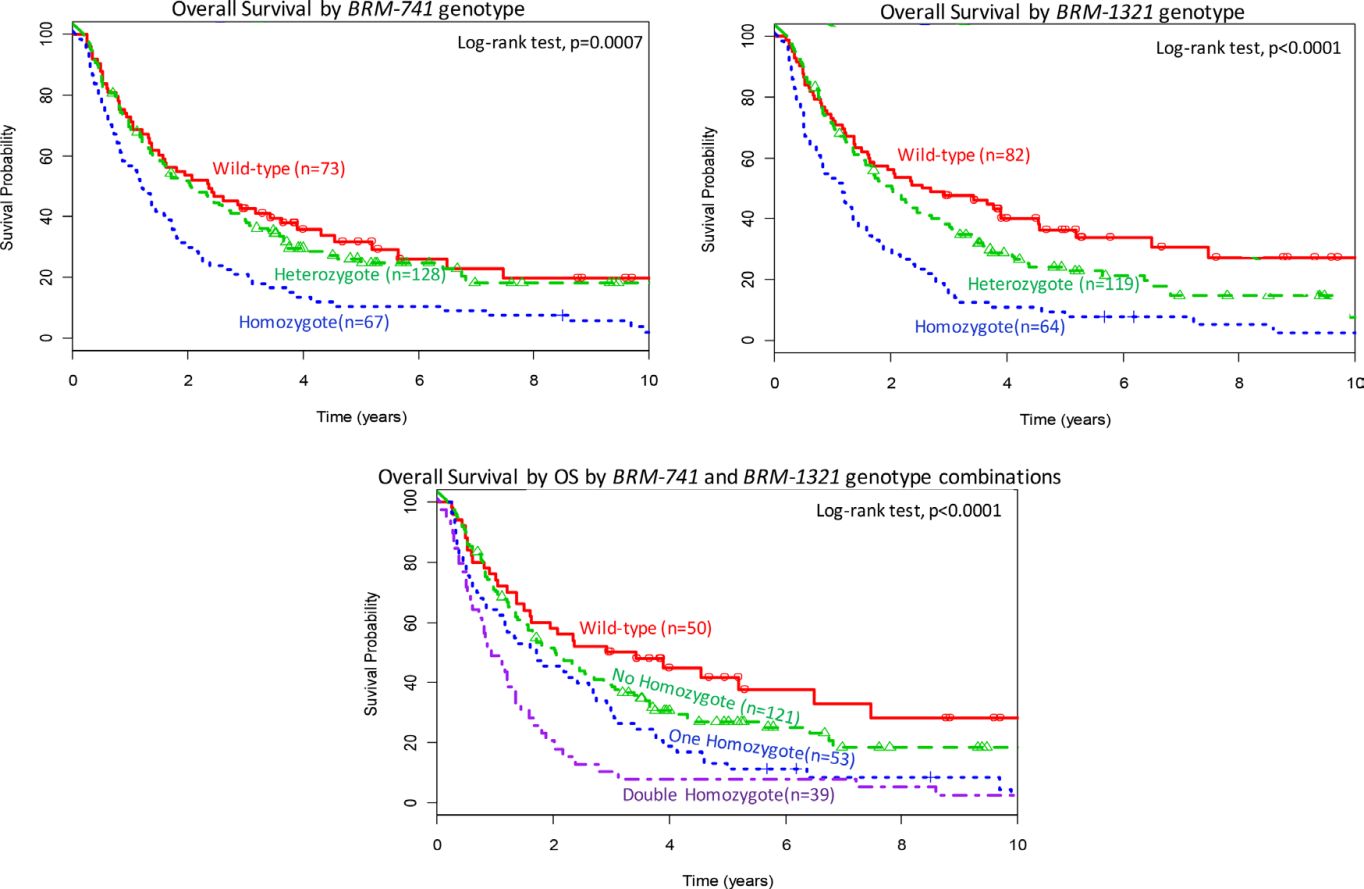
Overall survival (OS) by BRM polymorphism status Top panel: OS by *BRM-741* genotype; Middle panel: OS by *BRM-1321* genotype; Bottom panel: OS by a combination of *BRM-741* and *BRM-1321* genotypes.

In Kaplan Meier curves (Figure 1) and in univariable and multivariable Cox models (Table 3 and Supplementary Table 1), Homozygous variants of *BRM-741* were associated strongly with worse OS (adjusted HR [aHR] 1.64; 95% CI: 1.1–2.4; *p* = 0.0026] and with worse PFS (aHR 1.65; 95% CI: 1.1–2.4; *p* = 0.009), when each was compared to the wild-type genotype. Similar findings were observed for the *BRM-1321* homozygous variants for OS (aHR 2.09; 95% CI: 1.4–3.0; *p* = 0.0005) and PFS 2.29; 95% CI: 1.6–3.4; *p* < 0.0001). Carrying the double homozygous *BRM-741* and *BRM-1321* variants was associated strongly with substantially worse OS (aHR 2.21; 95% CI:1.4–3.6, *p* < 0.0001) and PFS (aHR 2.47; 95% CI: 1.5–4.0, *p* < 0.0001), when compared to the double wild-type genotypes. Though there was no statistical power to make firm conclusions, an exploratory interaction analysis of the two polymorphisms was highly insignificant (interaction term, theta = 1.05 (95% 0.24–6.33); *p* = 0.74), and suggests an additive effect of each polymorphism on survival.

**Table 3:** Association between *BRM* polymorphisms and esophageal adenocarcinoma overall survival

Comparisons	Univariable Analysis			Multivariable analysis*		
	HR	95% CI	*p*-value	HR	95% CI	*p*-value
*BRM-741* polymorphism						
Heterozygote vs Wild-type	1.12	0.79–1.57	0.53	1.07	0.76–1.5	0.71
Homozygote vs Wild-type	1.87	1.29–2.70	0.0012	1.64	1.13–2.37	0.0026
per increase in one minor allele**	1.34	1.14–1.89	0.01	1.30	1.06–1.84	0.03
*BRM-1321* polymorphism						
Heterozygote vs Wild-type	1.38	0.98–1.93	0.063	1.37	0.97–1.94	0.075
Homozygote vs Wild-type	2.41	1.66–3.49	0.0003	2.09	1.43–3.04	0.0005
per increase in one minor allele**	1.47	1.26–2.04	0.008	1.38	1.15–1.97	0.02
*Both Polymorphisms*						
No homozygote vs Double Wild-type	1.38	0.92–2.08	0.13	1.25	0.82–1.88	0.30
One homozygote vs Double Wild-type	1.98	1.27–3.11	0.003	1.82	1.15–2.89	0.011
Double homozygote vs Double Wild-type	2.97	1.84–4.79	< 0.0001	2.21	1.36–3.58	0.0004
per increase in one minor allele**	1.32	1.11–1.76	0.002	1.28	1.12–1.72	0.003

*After adjustment to variables included in clinical base model (age, ECOG performance status, weight loss (%), TNM Classification of disease stage at diagnosis, and surgical resection); HR, hazard ratio; CI, Confidence Interval; vs, versus; Note that radiation therapy was highly collinear with disease stage at diagnosis (R = 0.73, *p* < 0.0001) and was therefore not included as a final adjustment variable); ** additive inheritance genetic model.

Subgroup analyses by median age (> median, ≤ median), sex (female, male), smoking status (current smoker, former, current smoker), ethnicity (Caucasian *vs*. other),, performance status (0 *vs*. 1), lifetime alcohol use (Yes *vs*. No) tumour location (distal esophageal, gastroesophageal cancer), presence or history of Barrett's esophagus (Yes, No), use of platinum agents in the initial treatment (Yes, No), primary surgical resection (Yes, No), use of radiation therapy in initial treatment (Yes, No) and disease stage (Stages I-III, IV) were all consistent with the primary result, with numerical aHRs of the double homozygous variants, when compared to double wild-type genotypes, of between 1.63–3.44 for OS and 1.78–3.52 for PFS; with these subgroup analyses, significance was not reported specifically because the goal was to evaluate consistency of magnitude and direction of association (Supplementary Table 2 summarizes results of the additive genetic inheritance model).

## DISCUSSION

In this study we report that *BRM-741* and *BRM-1321* polymorphisms are independent prognostic factors for EAC outcome in our dataset. This significant association was observed irrespective of clinico-demographic variables. The recent marked increase in EAC incidence in developed countries and its poor prognosis and treatment outcome represent a substantial health problem. While the etiologies for the increased EAC incidence is largely unknown, whole genome sequencing studies identified new genomic alterations that may contribute to EAC growth and progression, including chromatin remodeling factors like *BRM*, that are SWI/SNF family members. [7] Liu et al. previously demonstrated that the double homozygous *BRM* variants increased the risk of all stages of lung cancer among ever-smokers, with adjusted odds ratio [aOR] of 2.19; 95% CI, 1.4–3.4; *P* = 0.0006). [20] This finding was further confirmed in a study of early-stage lung cancer patients, where the double homozygotes more than doubled significantly lung cancer risk (aOR, 2.61; *p* = 0.006), [21] and in a separate assessment of head and neck cancers (aOR, 2.75; *P* = 0.004). However, there was only a trend towards increased risk of esophageal cancer in double *BRM* homozygotes (aOR, 1.66; *P* = 0.31, when compared to the double wildtype genotype). However, this esophageal cancer analyses did not have adequate power to evaluate EAC separately from esophageal squamous cell carcinoma; [21] We recently increased the sample size of EACs to 270 and compared it to matched controls; *BRM* variants continued to have an aOR of EAC risk of around 1.7 (*p* = 0.09; unpublished). An association between other *BRM* genetic variants and hepatocellular carcinoma (HCC) risk was also reported in two separate case-control studies in Chinese patients (though those specific genetic variants appeared to be restricted mainly to patients of East Asian descent). [24] The totality of data suggest that these *BRM* promoter polymorphisms have the ability to regulate *BRM* expression, and these genetic susceptibility markers are associated with increased risk of multiple different cancer types.

As chromatin remodeling pathways can influence carcinogenesis, so may the biology of the subsequent cancers influence prognosis, either through being more or less biologically aggressive, or through response to therapy. We identified this in pancreatic cancer patients recently. [22] As the majority of patients received radiation and chemotherapy, we were unable to ascertain whether the impact of these polymorphisms on survival outcome is mediated through aggressive biology or through specific treatment modalities. A sub-analysis of patients treated by cisplatin-based chemotherapy *versus* not also found no substantial differences in the outcome relationships with *BRM* polymorphisms. However, the ability of these promoter polymorphisms to lead to suppression of BRM function through decreased gene expression is of great potential clinical relevance. [20] Our study contributes to the growing evidence that the *BRM* polymorphisms are strong germline biomarkers that may select out a subset of high risk patients that are less likely to develop cancer, and another subset with poor prognoses that fail standard treatments.

In addition, there are potential treatment implications in finding an association between the *BRM* promoter variants and EAC outcome. Gramling *et al*. demonstrated that drug-induced recovery of BRM expression and function in several BRM-deficient cell lines can occur when two novel potentially effective agents were identified from a high-throughput drug screen. [20, 23, 24] Further, the double homozygous variants resulted in the epigenetic loss of BRM expression in dozens of cancer cell lines and primary lung and HCC tumors. Flavonoids may also reverse these epigenetic loss of expression. [25, 26] Although epigenetic BRM silencing has yet to be proven as an effective oncogenic driver, the current data suggest that reversing epigenetic dysregulation can lead to a novel preventive and therapeutic approaches in selected solid malignancies. The double homozygotes were found to have a significantly worse outcome compared to the heterozygotes or single homozygotes; a gene-dose effect was suggested by gradient values. Perhaps the repression of BRM only occurs strongly when both homozygous insertion alleles are present. Loss of heterozygosity is common in the 9q23–24 region, and selectively losing the wild-type deletion alleles during carcinogenesis may lead poor outcome in individuals carrying the germline heterozygotes in one or both polymorphisms, as seen in the current and prior studies. [20] Additional molecular studies are necessary to evaluate the consequences of using BRM expression or these BRM promoter genotypes and their mechanisms in promoting cancer risk and outcome. Perhaps flavonoids could also be piloted as adjunctive therapy in Stage I–III EAC patients carrying the double-homozygous variants.

Of several study limitations, firstly, the small number of EAC patients did not allow us to develop both training and validation datasets. Caucasians comprised most of the study population that were treated at a single institution; the study was of cross-sectional design. The study population was observational and included all stages, although we tried to use clinically-directed eligibility criteria to perform sensitivity analyses of smaller subsets demonstrating consistency across different subgroups, including stage-specific analyses. Nonetheless, findings will require validation in other patient datasets and in future studies. Our sample slightly under-represented metastatic adenocarcinoma patients, as only 35% of such patients were Stage IV at diagnosis; however, subgroup analyses found similar relationships by stage and histology. Finally, the proportion of patients with Barrett's esophagus was likely under-reported: one-third of patients did not have surgical resection and detection Barrett's esophagus may be difficult endoscopically in the presence of cancer in the same region; also the majority of tumors were located in areas as Barrett's esophagus, and these tumors may have grown over and masked the former presence of Barrett's esophagus.

In summary, two promoter *BRM* germline variants were associated with worse outcome in our esophageal adenocarcinoma (EAC) patients. This significantly poorer outcome was independent of TNM classification at diagnosis or other clinic-demographic variables. However, these promoter polymorphisms may contribute independently to cancer outcome. Additional studies can improve our understanding of *BRM* promoter and carcinogenesis.

## MATERIALS AND METHODS

The study population consisted of 270 adult patients (aged 18 years or older) with histologically confirmed esophageal adenocarcinoma or gastroesophageal cancer (Siewert I/II only) of all TNM stages treated in Princess Margaret (PM) Cancer Centre in Toronto from 2006 to 2013. All patients eligible for this analysis were deemed fit for standard stage-specific treatment by their clinicians, were cognitively able to consent, had decided to receive therapy at PM, and had a clinician-determined life expectancy of at least 12 weeks. Patients were seen in a multi-disciplinary fashion, and both random and difficult cases were reviewed by the entire team in weekly multidisciplinary rounds. Stage-specific treatment for localized disease was complete open surgical resection; the rare patient (*n* = 3) undergoing robotic, endoscopic, or laparoscopic resection was excluded. Stage-specific treatment for locoregional disease (i.e., metastatic locoregional lymph nodes) was treated either with chemoradiation or trimodality (neoadjuvant chemoradiation) therapy; neoadjuvant chemotherapy without radiation was reserved for cases where radiation was contraindicated due to either co-morbidity or requirement of radiation doses that exceed allowable safety margins for normal tissue tolerance. Stage-specific treatment for Stage IV (distant disease) included palliative chemotherapy (oral or intravenous, single agent or combination chemotherapy), although sequential or concurrent radiation could also be used for symptomatic management of patients. The EACs in this study were treated during the era when HER2 testing was neither performed standardly nor was Herceptin used (it was evaluated and used for gastric cancer, though). Staging was performed using the 7th edition of the TNM Staging system (older cases were re-staged). Anatomic location was based on the midpoint of the lesion; when there was uncertainty as to whether the patient had esophageal adenocarcinoma or Siewert I–II gastroesophageal vs gastric or Siewert III gastroesophageal adenocarcinoma, these cases were excluded from analysis. Mixed histologic subtypes (e.g. sarcoma and adenocarcinoma) were excluded from analysis.

Demographic and epidemiological data were collected prospectively using a standardized questionnaire, and stored in ACCESS database. Clinico-pathological and treatment data were extracted from the patients’ medical records. Overall survival data were obtained from a combination of the Ontario Cancer Registry, Hospital Cancer Registry, patient/family, palliative care, and referring physician phone contact. The date of death or date last known to be alive was used for overall survival analysis. For the exploratory endpoint of date of first progressive disease, medical charts were used to determine the radiological date of progression; when no radiology was performed, the date that the clinician classified the patient has having clinically progressive disease was used. Dates of last assessment that found no evidence of first progression was used for censoring purposes in the progression-free exploratory analysis.

Genomic DNA was extracted from whole blood-derived lymphocytes of the 270 patients with EAC according to previously described protocols. [20] Genotyping of the *BRM-741* and *BRM-1321* promoter insertion polymorphisms was conducted on extracted DNA by qPCR using TaqMan probes (Life Technologies Inc., Burlington, Canada). The primers and PCR protocol used have been described previously [20].

Association of the polymorphisms with overall (OS) and the exploratory progression free survival (PFS) were assessed using multivariable Cox proportional hazards models. Variables assessed in the multivariable model were based on clinical knowledge: age, gender, ECOG performance status, presence of weight loss at diagnosis, Body Mass Index (BMI), alcohol use, TNM classification, presence of Barrett's esophagus, type of received treatment (surgery, cisplatin-based chemotherapy, radiation). Clinico-demographic variables significant in univariable analyses were included in a backwards selection procedure that resulted in a clinical base multivariable analysis model. Then after forcing age and gender back into the model, the variables in this new base model was used to adjust for the association between *BRM* polymorphisms and survival outcomes. Categorizations used for the various levels of the two *BRM* polymorphisms were based on prior knowledge of putative BRM polymorphism function and prior associations with risk/outcome. [20–24]

## SUPPLEMENTARY MATERIALS TABLES



## Abbreviations

aHR: Adjusted Hazard Ratio
aOR: Adjusted Odds Ratio
ATP: Adenosine triphosphate
BMI: Body Mass Index
BRM: Brahma
CI: Confidence Interval
DNA: Deoxyribonucleic acid
EAC: Esophageal adenocarcinoma
ECOG: Eastern Cooperative Oncology Group
HCC: Hepatocellular carcinoma
HER2: Human epidermal growth factor receptor 2
LOH: Loss of heterozygosity
OS: Overall Survival
PCR: Polymerase chain reaction
PFS: Progression free survival
PM: Princess Margaret
qPCR: Quantitative polymerase chain reaction
SWI: SWItch
SNF: Sucrose non-fermentable
TNM: Cancer staging (tumour, lymph nodes, metastasis)

## References

[1] Holmes RS, Vaughan TL (2007). Epidemiology and pathogenesis of esophageal cancer. Semin Radiat Oncol.

[2] Pohl H, Welch HG (2005). The role of overdiagnosis and reclassification in the marked increase of esophageal adenocarcinoma incidence. J Natl Cancer Inst.

[3] Devesa SS, Blot WJ, Fraumeni JF (1998). Changing patterns in the incidence of esophageal and gastric carcinoma in the United States. Cancer.

[4] Blot WJ, McLaughlin JK (1999). The changing epidemiology of esophageal cancer. Semin Oncol.

[5] Lagergren J, Bergstrom R, Lindgren A, Nyren O (1999). Symptomatic gastroesophageal reflux as a risk factor for esophageal adenocarcinoma. N Engl J Med.

[6] Rustgi AK, El-Serag HB (2014). Esophageal carcinoma. N Engl J Med.

[7] Dulak AM, Stojanov P, Peng S, Lawrence MS, Fox C, Stewart C, Bandla S, Imamura Y, Schumacher SE, Shefler E, McKenna A, Carter SL, Cibulskis K (2013). Exome and whole-genome sequencing of esophageal adenocarcinoma identifies recurrent driver events and mutational complexity. Nat Genet.

[8] Song Y, Li L, Ou Y, ao Z, Li E, Li X, Zhang W, Wang J, Xu L, Zhou Y, Ma X, Liu L, Zhao Z (2014). Identification of genomic alterations in oesophageal squamous cell cancer. Nature.

[9] Weaver JM, Ross-Innes CS, Shannon N, Lynch AG, Forshew T, Barbera M, Murtaza M, Ong CA, Lao-Sirieix P, Dunning MJ, Smith L, Smith ML, Anderson CL (2014). Ordering of mutations in preinvasive disease stages of esophageal carcinogenesis. Nat Genet.

[10] Wang W, Cote J, Xue Y, Zhou S, Khavari PA, Biggar SR, Muchardt C, Kalpana GV, Goff SP, Yaniv M, Workman JL, Crabtree GR (1996). Purification and biochemical heterogeneity of the mammalian SWI-SNF complex. Embo J.

[11] Muchardt C, Yaniv M (2001). When the SWI/SNF complex remodels the cell cycle. Oncogene.

[12] Reisman D, Glaros S, Thompson EA (2009). The SWI/SNF complex and cancer. Oncogene.

[13] Phelan ML, Sif S, Narlikar GJ, Kingston RE (1999). Reconstitution of a core chromatin remodeling complex from SWI/SNF subunits. Mol Cell.

[14] Glaros S, Cirrincione GM, Muchardt C, Kleer CG, Michael CW, Reisman D (2007). The reversible epigenetic silencing of BRM: implications for clinical targeted therapy. Oncogene.

[15] An HX, Claas A, Savelyeva L, Seitz S, Schlag P, Scherneck S, Schwab M (1999). Two regions of deletion in 9p23–24 in sporadic breast cancer. Cancer Res.

[16] Girard L, Zochbauer-Muller S, Virmani AK, Gazdar AF, Minna JD (2000). Genome-wide allelotyping of lung cancer identifies new regions of allelic loss, differences between small cell lung cancer and non-small cell lung cancer, and loci clustering. Cancer Res.

[17] Sarkar S, Roy BC, Hatano N, Aoyagi T, Gohji K, Kiyama R (2002). A novel ankyrin repeat-containing gene (Kank) located at 9p24 is a growth suppressor of renal cell carcinoma. J Biol Chem.

[18] Gunduz E, Gunduz M, Ali MA, Beder L, Tamamura R, Katase N, Tominaga S, Yamanaka N, Shimizu K, Nagatsuka H (2009). Loss of heterozygosity at the 9p21–24 region and identification of BRM as a candidate tumor suppressor gene in head and neck squamous cell carcinoma. Cancer Invest.

[19] Wang JR, Gramling SJ, Goldstein DP, Cheng D, Chen D, Azad AK, Tse A, Hon H, Chen Z, Mirshams M, Simpson C, Huang SH, Marquez S (2013). Association of two BRM promoter polymorphisms with head and neck squamous cell carcinoma risk. Carcinogenesis.

[20] Liu G, Gramling S, Munoz D, Cheng D, Azad AK, Mirshams M, Chen Z, Xu W, Roberts H, Shepherd FA, Tsao MS, Reisman D (2011). Two novel BRM insertion promoter sequence variants are associated with loss of BRM expression and lung cancer risk. Oncogene.

[21] Wong KM, Qiu X, Cheng D, Azad AK, Habbous S, Palepu P, Mirshams M, Patel D, Chen Z, Roberts H, Knox J, Marquez S, Wong R (2014). Two BRM promoter insertion polymorphisms increase the risk of early-stage upper aerodigestive tract cancers. Cancer Med.

[22] Segedi M, Anderson LN, Espin-Garcia O, Borgida O, Bianco T, Cheng D, Chen Z, Patel D, Brown MC, Xu W, Reisman DR, Gallinger S, Cotterchio M BRM polymorphisms, pancreatic cancer risk and survival. Int J Cancer (in presss).

[23] Gramling S, Rogers C, Liu G, Reisman D (2011). Pharmacologic reversal of epigenetic silencing of the anticancer protein BRM: a novel targeted treatment strategy. Oncogene.

[24] Gao X, Huang M, Liu L, He Y, Yu Q, Zhao H, Zhou C, Zhang J, Zhu Z, Wan J, Jiang X, Gao Y (2013). Insertion/deletion polymorphisms in the promoter region of BRM contribute to risk of hepatocellular carcinoma in Chinese populations. PLoS One.

[25] Kahali B, Yu J, Marquez SB, Thompson KW, Liang SY, Lu L, Reisman D (2014). The silencing of the SWI/SNF subunit and anticancer gene BRM in Rhabdoid tumors. Oncotarget.

[26] Kahali B, Marquez SB, Thompson KW, Yu J, Gramling SJ, Lu L, Aponick A, Reisman D (2014). Flavonoids from each of the six structural groups reactivate BRM, a possible cofactor for the anticancer effects of flavonoids. Carcinogenesis.

