# Supplementary material for: Association of BRM promoter polymorphisms and esophageal adenocarcinoma outcome
Source: Oncotarget. 2017 Mar 3;8(17):28093–100. doi: 10.18632/oncotarget.15890 (PMC5438633; doi:10.18632/oncotarget.15890)
Supplement: Supplementary file 1 [file oncotarget-08-28093-s001.pdf]

Association of BRM promoter polymorphisms and esophageal adenocarcinoma outcome

Supplementary Materials

Supplementary Table 1: Association between *BRM* polymorphisms and esophageal adenocarcinoma progression-free survival

Variables	Univariable Analysis			Multivariable analysis*		
	HR	95% CI	*p*–value	HR	95% CI	*p*–value
BRM-741 polymorphism**						
Heterozygous	1.07	0.83–1.50	0.68	1.06	0.76–1.49	0.72
Homozygous	1.69	1.18–2.50	0.0053	1.65	1.13–2.39	0.009
per increase in one minor allele**	1.28	1.06–1.65	0.006	1.29	1.04–1.74	0.004
BRM-1321 polymorphism**						
Heterozygous	1.36	0.97–1.89	0.071	1.52	1.08–2.14	0.017
Homozygous	2.25	1.56–3.25	< 0.0001	2.29	1.56–3.35	< 0.0001
per increase in one minor allele**	1.36	1.18–1.68	0.0006	1.38	1.20–1.72	0.0002
BRM-741/1321 polymorphism***						
None homozygous	1.32	0.89–1.97	0.17	1.28	0.86–1.91	0.23
One homozygous	1.64	1.05–2.56	0.031	1.64	1.03–2.61	0.036
Two homozygous	2.84	1.77–4.56	< 0.0001	2.47	1.53–3.98	< 0.0001
per increase in one minor allele**	1.32	1.14–1.72	0.0002	1.33	1.19–1.63	< 0.0001

*After adjustment of variables included in clinical base model (age, gender, ECOG, weight loss (%), TNM Classification at diagnosis, and surgical resection (Yes/No)) **reference category is the wildtype; ***reference category is the double-wildtype.

Supplementary Table 2: Subgroup analysis between the association between *BRM* polymorphisms and esophageal adenocarcinoma overall survival

Subgroup Categories	Adjusted hazard ratio per increase in each minor allele		
	BRM-741 polymorphism	*BRM-1321* polymorphism	Combined *BRM-741/BRM-1321* polymorphisms
All patients	1.30	1.38	1.28
Median age or older	1.19	1.46	1.30
Less than Median age	1.43	1.28	1.27
Male	1.36	1.42	1.32
Female	1.22	1.33	1.25
Caucasian	1.24	1.43	1.34
Other	1.40	1.18	1.24
Never smoker	1.53	1.50	1.43
Former smoker	1.09	1.43	1.22
Current smoker	1.26	1.06	1.22
Performance status ECOG 0*	1.43	1.36	1.29
Performance status ECOG 1*	1.23	1.45	1.29
Lifetime alcohol use: Yes	1.26	1.15	1.24
Lifetime alcohol use: No	1.34	1.46	1.36
Distal esophageal location*	1.21	1.43	1.32
Gastroesophageal junction location*	1.35	1.40	1.30
Presence of Barrett's Esophagus	1.34	1.26	1.32
Absence of Barrett's Esophagus	1.29	1.43	1.22
Platinum Agent given	1.17	1.55	1.35
No platinum agent given	1.43	1.29	1.27
Surgical Resection	1.24	1.30	1.24
No surgical resection	1.53	1.55	1.43
Radiation given	1.18	1.62	1.31
No radiation given	1.36	1.24	1.28
Disease Stage I-III	1.25	1.34	1.24
Disease Stage	1.50	1.52	1.42

The additive genetic inheritance model is presented, after adjustment of variables included in clinical base model (age, gender, ECOG, weight loss (%), TNM Classification at diagnosis, and surgical resection (Yes/No), where relevant). Given the small sample sizes of some of the subgroups, and that the purpose of this analysis was to assess consistency in direction and magnitude of associations, only the numeric value is given without confidence intervals and without significance testing.
*There were too few individuals in some subgroup categories to generate a stable estimate.
